# Establishment of the MID‐NET^®^ medical information database network as a reliable and valuable database for drug safety assessments in Japan

**DOI:** 10.1002/pds.4879

**Published:** 2019-08-29

**Authors:** Mitsune Yamaguchi, Satomi Inomata, Sayoko Harada, Yu Matsuzaki, Maiko Kawaguchi, Mayuko Ujibe, Mari Kishiba, Yoshiaki Fujimura, Michio Kimura, Koichiro Murata, Naoki Nakashima, Masaharu Nakayama, Kazuhiko Ohe, Takao Orii, Eizaburo Sueoka, Takahiro Suzuki, Hideto Yokoi, Fumitaka Takahashi, Yoshiaki Uyama

**Affiliations:** ^1^ Office of Medical Informatics and Epidemiology Pharmaceuticals and Medical Devices Agency Tokyo Japan; ^2^ Head office Tokushukai Information System Incorporated Osaka Japan; ^3^ Department of Medical Informatics Hamamatsu University Hospital Shizuoka Japan; ^4^ Department of Radiology Kitasato University Hospital Kanagawa Japan; ^5^ Department of Advanced Information Technology Kyushu University Hospital Fukuoka Japan; ^6^ Medical IT Center Tohoku University Hospital Sendai Japan; ^7^ Department of Healthcare Information Management The University of Tokyo Hospital Tokyo Japan; ^8^ Department of Pharmacy NTT Medical Center Tokyo Tokyo Japan; ^9^ Department of Laboratory Medicine Saga University Hospital Saga Japan; ^10^ Department of Medical Informatics Chiba University Hospital Chiba Japan; ^11^ Department of Medical Informatics Kagawa University Hospital Kagawa Japan

**Keywords:** data quality, drug safety, medical information database, MID‐NET^®^, real‐world data, regulatory science

## Abstract

**Purpose:**

To establish a new medical information database network (designated MID‐NET^®^) to provide real‐world data for drug safety assessments in Japan.

**Methods:**

This network was designed and developed by the Ministry of Health, Labour and Welfare and the Pharmaceuticals and Medical Devices Agency in collaboration with 23 hospitals from 10 healthcare organizations across Japan. MID‐NET^®^ is a distributed and closed network system that connects all collaborative organizations through a central data center. A wide variety of data are available for analyses, including clinical and administrative information. Several coding standards are used to standardize the data stored in MID‐NET^®^ to allow the integration of information originating from different hospitals. A rigorous and consistent quality management system was implemented to ensure that MID‐NET^®^ data are of high quality and meet Japanese regulatory standards (good post‐marketing study practice and related guidelines).

**Results:**

MID‐NET^®^ was successfully established as a reliable and valuable medical information database and was officially launched in April 2018. High data quality with almost 100% consistency was confirmed between original data in hospitals and the data stored in MID‐NET^®^. A major advantage is that approximately 260 clinical laboratory test results are available for analysis.

**Conclusions:**

MID‐NET^®^ is expected to be a major data source for drug safety assessments in Japan. Experiences and best practices established in MID‐NET^®^ may provide a model for the future development of similar database networks.

**Key Points**

- MID‐NET^®^ is a new medical information database network created by Japan's Ministry of Health, Labour and Welfare and the Pharmaceuticals and Medical Devices Agency in collaboration with 23 hospitals from 10 healthcare organizations.
- The database encompasses a wide variety of data, including clinical and administrative information.
- MID‐NET^®^ also includes standardized data of test results from approximately 260 laboratory tests (as of December 2018) that can be used as clinical indicators in drug safety assessments.
- The high quality of MID‐NET^®^ data is ensured using various quality management systems, including the daily monitoring of messages and periodic data consistency checks.
- MID‐NET^®^ is expected to be a major data source for drug safety assessments in Japan.

## 1 INTRODUCTION

The utilization of real‐world data for regulatory purposes has been actively discussed and pursued in recent years.^1^, ^2^, ^3^ For example, the US Food and Drug Administration established the Sentinel Initiative in 2008 to explore the creation of an electronic system to monitor the safety of its regulated products.^4^, ^5^

**Figure 1:**
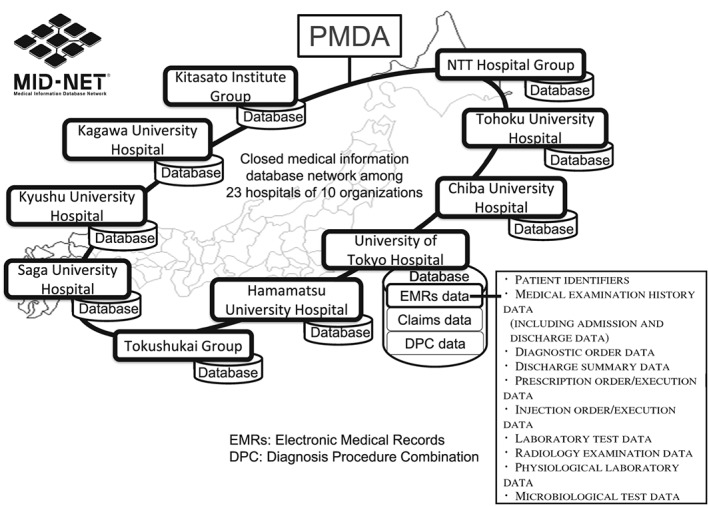
Partner hospitals and data categories of MID‐NET^®^. MID‐NET^®^ is a database network established by Japan's Ministry of Health, Labour and Welfare and the Pharmaceuticals and Medical Devices Agency (PMDA) in collaboration with 23 hospitals from 10 healthcare organizations. MID‐NET^®^ includes hospital information system (HIS) data such as electronic medical records (EMRs), claims data, and diagnosis procedure combination (DPC) data. Note that radiology examination data and physiological laboratory data only include order and execution data but not results such as images

In 2011, Japan's Ministry of Health, Labour and Welfare (MHLW) and the Pharmaceuticals and Medical Devices Agency (PMDA) started an initiative to establish a medical information database network (designated MID‐NET^®^) that would enable the utilization of real‐world data for drug safety assessments.^6^ This initiative involved collaborations with 23 hospitals from 10 healthcare organizations across Japan (Chiba University Hospital, Hamamatsu University Hospital, Kagawa University Hospital, four hospitals from the Kitasato Institute Group, Kyushu University Hospital, Tohoku University Hospital, 10 hospitals from the Tokushukai Medical Group, two hospitals from the NTT Hospital Group, Saga University Hospital, and the University of Tokyo Hospital). These partner hospitals were selected from an open recruitment requesting for cooperation with the MID‐NET^®^ project. Figure 1 presents an overview of the partner hospitals and the types of data stored in the MID‐NET^®^ system. All expenses for this project were financed from a government budget of the MHLW and the PMDA's own budget, which was originally derived from contributions from the pharmaceutical industry for the purpose of developing safety measures.

MID‐NET^®^ was officially launched on April 1, 2018, whereupon the database network became available to analysts in the pharmaceutical industry and academia. Prior to launch, the database had only been used by the MHLW, the PMDA, and the collaborative organizations. MID‐NET^®^ is anticipated to become a major data source for clinical research, post‐marketing drug safety studies conducted by the pharmaceutical industry, and drug safety assessments conducted by the PMDA under the MIHARI framework.^1^

In this article, we discuss how MID‐NET^®^ was established, and share our experiences in creating a reliable and valuable database to enable accurate assessments of drug safety and promote the utilization of real‐world data in regulatory decision‐making.

## 2 OVERVIEW OF THE MID‐NET^®^ SYSTEM

MID‐NET^®^ adopts a common data model that stores a wide variety of hospital information system (HIS) data (Figure 1) such as electronic medical records (EMRs), administrative claims data, and diagnosis procedure combination (DPC) data.^7^, ^8^ EMRs constitute a particularly important component of the MID‐NET^®^ system and are standardized based on the message specifications of SS‐MIX2.^7^ EMRs include different types of information, such as patient identifiers, medical examination history data (including admission and discharge data), diagnostic orders data, discharge summary data, prescription orders/execution data, injection orders/execution data, and laboratory test data. Administrative claims data are produced to determine reimbursements for inpatient and outpatient care according to a fee‐for‐service system. DPC data are produced to determine reimbursements for inpatient care according to diagnosis‐related groups, and provide important context in terms of patient case‐mix.

**Figure 2:**
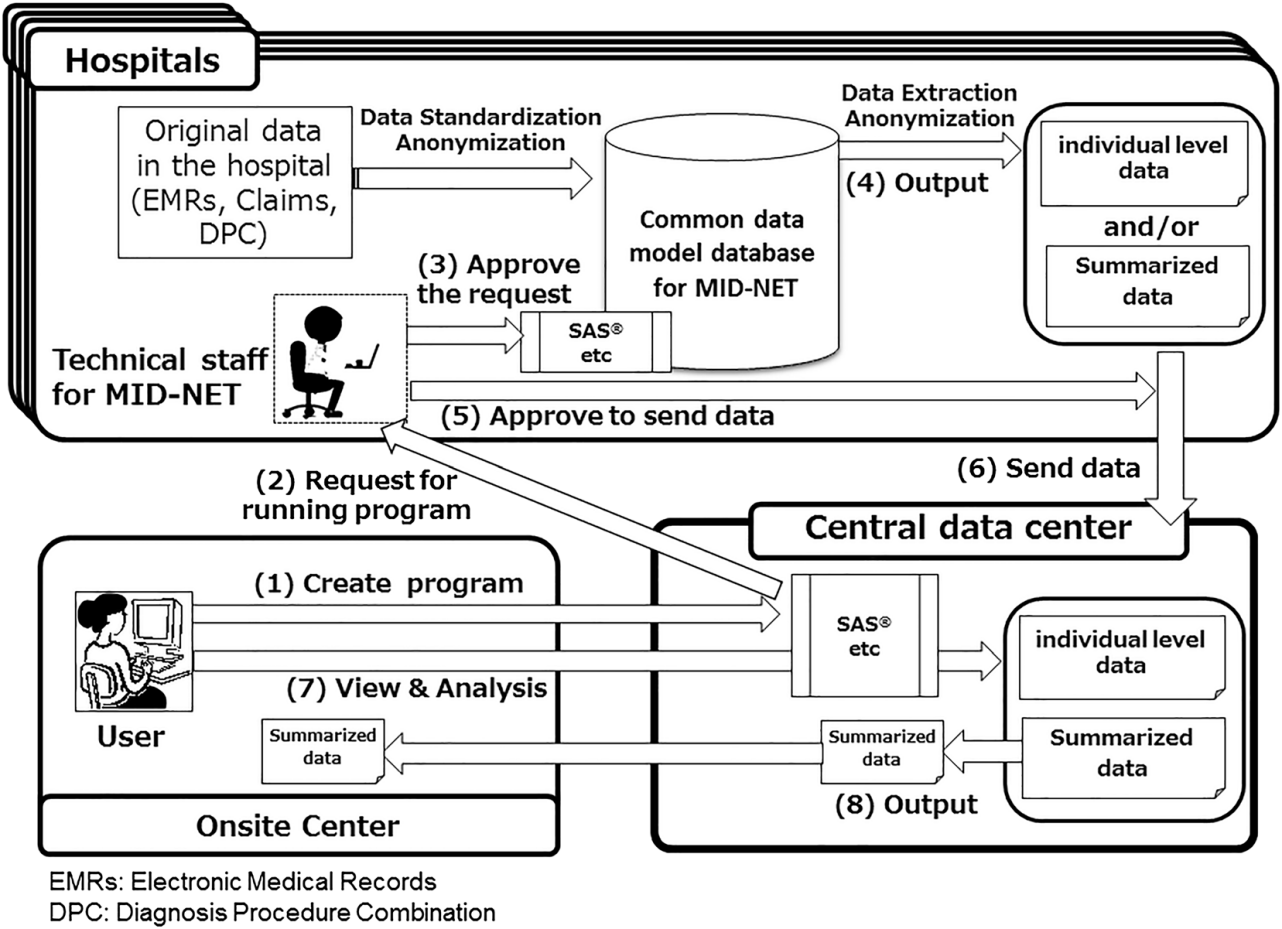
Outline of the MID‐NET^®^ system and the process of data extraction, transfer, and analysis

As shown in Figure 2, MID‐NET^®^ is a distributed and closed network system that connects all collaborative organizations through a central data center. The stored data are periodically updated (every week or 1‐3 months depending on the type of data) to provide access to the most up‐to‐date information from clinical practice. Analytical results are obtained through the following steps: (1) a user creates a program to extract and summarize the target data, such as data from patients who were prescribed a particular drug; (2) the user sends a request to approve the running of the program for analysis; (3) technical staff in the relevant collaborative organization approve the request (if applicable); (4) The program is used to extract the target data from MID‐NET^®^ and/or obtain summarized data; (5) technical staff in the relevant collaborative organization approve the sending of the extracted data (summarized and/or individual‐level data) to the central data center; (6) the extracted data are sent to the central data center; (7) the user remotely accesses the extracted data and conducts more detailed pharmacoepidemiological analyses using statistical programs such as SAS^®^ as required; (8) The user can locally access the summarized data after the analysis is completed, but cannot download individual‐level data. However, users are able to reaccess individual‐level data if necessary, as the data are stored and maintained in the central data center for a prespecified period of time (standard: 2 years; legally required study for a new molecular entity: 8 years and more to allow reexamination submissions).^9^

MID‐NET^®^ is operated and managed under the Act on PMDA (Act No. 192, 2002), and is exempt from requirements to obtain informed consent from patients in accordance with the Act on the Protection of Personal Information (Act No. 57, 2003). Nevertheless, in consideration of the characteristics of HIS data and ensuring transparency to patients, we have also undertaken the following measures: (a) the partner hospitals have affirmed that anonymized HIS data are utilized in MID‐NET^®^ and (b) the PMDA discloses information on the utilization of MID‐NET^®^ data and provides opportunities for patients to deny the provision of their HIS data to MID‐NET^®^. Furthermore, individual‐level data are automatically anonymized to protect patient privacy through the designation of new patient identification numbers, deletion of personally identifiable information (name, address, and residential postal code), and the random shifting of dates while maintaining the original intervals between dates (see Figure S1 for details of the data anonymization process). Thus, users are only able to access anonymized data for their analyses.

## 3 DATA QUALITY MANAGEMENT

The PMDA actively works with all collaborative organizations to ensure the quality of MID‐NET^®^ data, which is defined as ensuring that the original data from all partner hospitals are appropriately sent to and stored in MID‐NET^®^ in a standardized format (SS‐MIX2 [HL7‐based standard]^7^ for EMRs and the governmental reimbursement rules for administrative claims and DPC data) with high levels of accuracy, consistency, and completeness. In the real‐world setting, however, data patterns entered into hospital systems can vary even in cases where SS‐MIX2 is applied. MID‐NET^®^ also receives data on a daily basis from a variety of systems in each hospital in order to achieve timely updates, which is a notable feature of this database. For example, an EMR system can connect with several different specialized and independent clinical operating systems, including clinical laboratory testing, nursing, and radiology examination systems. Data in these systems are routinely sent through EMR systems to MID‐NET^®^. Because of the wide variations in hospital systems, it is very difficult to accurately anticipate all possible varieties of data messages during the system validation process. Furthermore, hospitals may implement configuration changes, modifications, or updates in one or more of these systems as part of improvements in daily clinical operations even after system reliability is confirmed. This further hinders the prediction of how such changes in hospital systems can affect MID‐NET^®^ data that are stored after secondary data collection. In addition, MID‐NET^®^ data are utilized in post‐marketing database studies that must comply with the quality standards stipulated in a ministerial ordinance for good post‐marketing study practice (GPSP)^10^ and their related guidelines.^11^ These regulations require the confirmation of database integrity in terms of data management and quality assurance (eg, accuracy, consistency, and completeness of data). Therefore, MID‐NET^®^ data quality cannot be ensured without daily and periodic monitoring with checks on actual data conditions. Similar practices for data quality assurance are implemented in the Sentinel Initiative's database, although daily management may not be required because of the lower frequency of updates (daily updates in the MID‐NET^®^ database vs periodic updates in the Sentinel database).^12^

**Figure 3:**
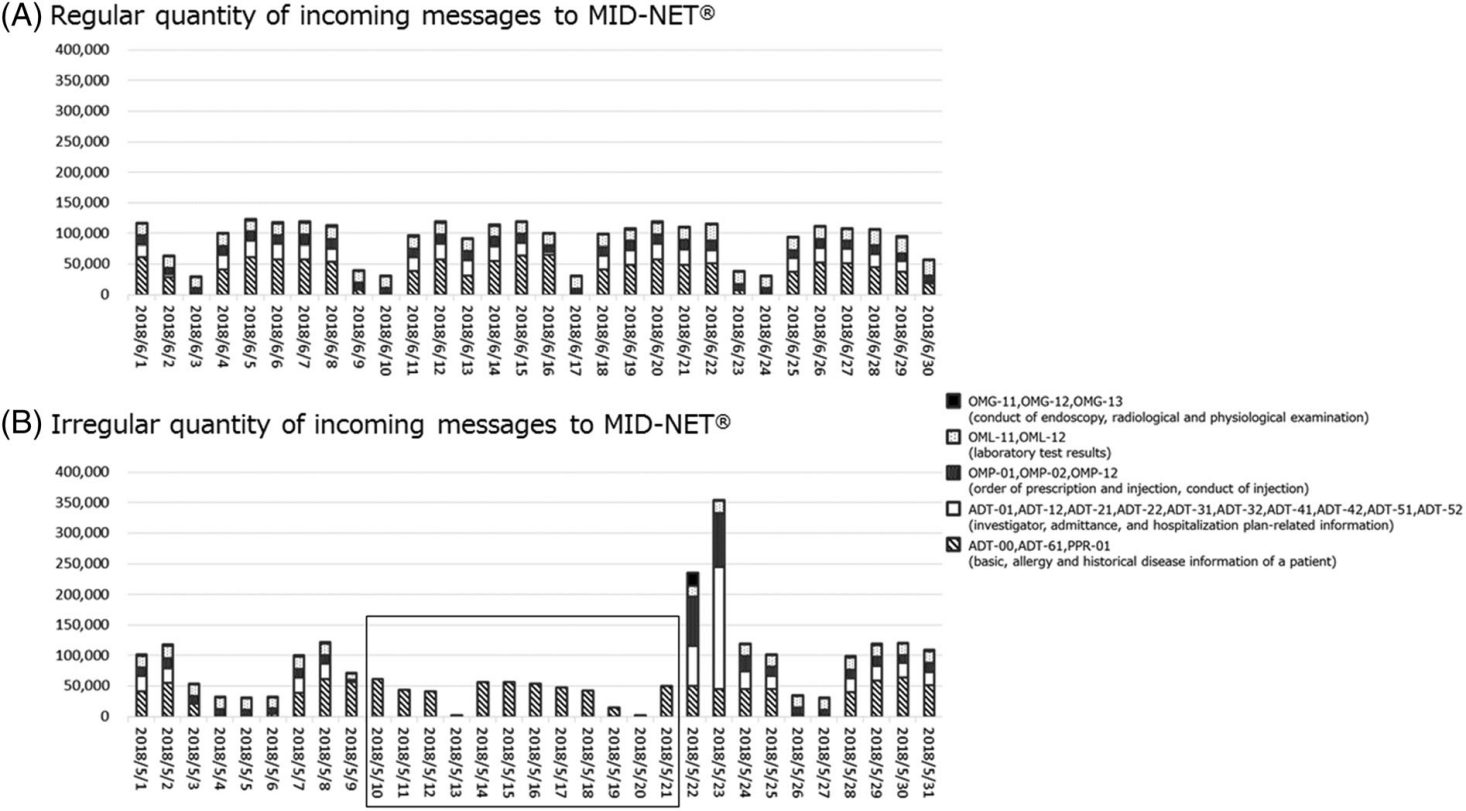
An example of daily quality management in MID‐NET^®^. A, Regular quantity of incoming messages to MID‐NET^®^ from a partner hospital. The quantity of incoming messages is generally consistent across weekdays, but lower on weekends. B, Irregular quantity of incoming messages to MID‐NET^®^ from a partner hospital. The box indicates a marked decrease in the number of incoming messages with several missing data elements (such as physiological examinations, laboratory test results, prescription orders, and hospitalization plan‐related information) over a 12‐day period (2018/5/10‐2018/5/21) because of an erroneous system setting. The missing messages were recovered over 2 days in the following week (2018/5/22‐2018/5/23) to avoid incomplete data storage. Note that the reduction in the number of messages on 2018/5/3 to 2018/5/4 was because of public holidays followed by the weekend

In daily quality management, data logs and the actual number of messages sent to MID‐NET^®^ are monitored (Figure 3). If any errors or marked changes in data size are detected, further investigations are conducted to identify the underlying reasons and to resolve any issues. For example, the quantity of incoming messages from one of the partner hospitals was found to be generally consistent on weekdays (Figure 3A). However, the daily monitoring system detected an irregular decrease in messages with several missing data elements over a 12‐day period (Figure 3B) because of an erroneous system setting. In this case, the PMDA promptly contacted the hospital and contractor, which immediately resolved the issue. Subsequently, the missing messages were recovered over 2 days in the following week to avoid incomplete data storage.

In addition to daily quality management, we also periodically check data completeness and consistency between original data in hospitals (EMRs, claims, and DPC data) and the data stored in MID‐NET^®^. The consistency checks are conducted through the following steps: (a) data are extracted for the target period (eg, 1 month) from both the original source and the MID‐NET^®^ database; (b) the consistency between these data is examined based on patient identification numbers, order numbers, and relevant dates (eg, drug administration start dates and laboratory test dates); (c) the underlying causes are identified if inconsistencies are detected; (d) appropriate measures (eg, fixing programs and resending the original data to MID‐NET^®^) are determined and implemented; and (e) resolution of the problem is verified. These steps are repeated until the restoration of consistency is confirmed. Several inconsistencies (eg, missing data and wrong data locations) were discovered in the initial stages of this project because of various reasons, including program errors, different interpretations of the SS‐MIX2 standard, and different health data management systems among the hospitals. Despite these issues, we were able to maintain high data quality with almost 100% consistency after implementing these quality management practices (Table 1). Although some inconsistencies are still occasionally observed, these are mainly because of a time‐lag between extracting the data and updating the information. At present, there appears to be no major reliability issues in MID‐NET^®^. Consistency checks are scheduled to be conducted for each partner hospital at least once a year to verify that no unexpected inconsistencies have occurred and to maintain the quality of MID‐NET^®^ data.

**Table 1:** Data consistency in major data categories in MID‐NET^®^

	Diagnostic Orders Data	Prescription Orders Data	Injection Orders Data	Laboratory Test Data
Chiba University Hospital	100.00% (30, 151/30, 151)	100.00% (104, 359/104, 359)	100.00% (141, 369/141, 369)	100.00% (1, 570, 704/1, 570, 704)
Hamamatsu University Hospital	100.00% (14, 782/14, 782)	100.00% (70, 505/70, 505)	100.00% (52, 546/52, 546)	100.00% (586, 482/586, 482)
Kagawa University Hospital	100.00% (11, 844/11, 844)	100.00% (56, 420/56, 420)	100.00% (64, 533/64, 533)	100.00% (366, 868/366, 868)
Kitasato Institute Group				
Kitasato University Hospital	100.00% (45, 863/45, 863)	100.00% (129, 227/129, 227)	100.00% (136, 201/136, 201)	100.00% (1, 166, 636/1, 166, 636)
Kitasato University East Hospital	100.00% (13, 256/13, 256)	100.00% (67, 498/67, 498)	100.00% (41, 237/41, 237)	100.00% (317, 189/317, 189)
Kitasato University Medical Center	100.00% (10, 769/10, 769)	100.00% (40, 102/40, 102)	100.00% (34, 199/34, 199)	100.00% (545, 350/545, 350)
Kitasato University Institute Hospital	100.00% (7,093/7,093)	100.00% (57, 649/57, 649)	100.00% (32, 411/32, 411)	100.00% (416, 525/416, 525)
Kyushu University Hospital	100.00% (40, 314/40, 314)	100.00% (128, 629/128, 629)	100.00% (148, 506/148, 506)	100.00% (1, 135, 766/1, 135, 766)
Tohoku University Hospital	100.00% (42, 893/42, 893)	100.00% (133, 953/133, 953)	100.00% (82, 859/82, 859)	100.00% (1, 287, 295/1, 287, 295)
Tokushukai Group				
Matsubara Tokushukai Hospital	100.00% (15, 192/15, 192)	100.00% (39, 980/39, 980)	100.00% (32, 336/32, 336)	100.00% (295, 427/295, 427)
Nozaki Tokushukai Hospital	99.98 % (16, 536/16, 539)	100.00% (39, 561/39, 561)	100.00% (29, 633/29, 633)	100.00% (214, 891/214, 891)
Kishiwada Tokushukai Hospital	100.00% (25, 801/25, 801)	100.00% (61, 039/61, 039)	100.00% (62, 975/62, 975)	100.00% (654, 823/654, 823)
Yao Tokushukai General Hospital	100.00% (21, 959/21, 959)	100.00% (71, 594/71, 594)	100.00% (53, 863/53, 863)	100.00% (587, 768/587, 768)
Fukuoka Tokushukai Hospital	100.00% (30, 867/30, 867)	100.00% (82, 205/82, 205)	100.00% (59, 081/59, 081)	100.00% (578, 625/578, 625)
Uji Tokushukai Hospital	100.00% (28, 668/28, 668)	100.00% (73, 325/73, 325)	100.00% (58, 347/58, 347)	100.00% (534, 720/534, 720)
Shonan Fujisawa Tokushukai Hospital	100.00% (32, 364/32, 364)	100.00% (59, 411/59, 411)	100.00% (43, 235/43, 235)	100.00% (603, 104/603, 104)
Sapporo Tokushukai Hospital	100.00% (15, 035/15, 035)	100.00% (53, 612/53, 612)	100.00% (35, 030/35, 030)	100.00% (230, 679/230, 679)
Nagoya Tokushukai General Hospital	100.00% (9, 342/9, 342)	100.00% (23, 007/23, 007)	100.00% (21, 706/21, 706)	100.00% (147, 253/147, 253)
Tokyo Nishi Tokushukai Hospital	100.00% (14, 607/14, 607)	100.00% (36, 119/36, 119)	100.00% (30, 052/30, 052)	100.00% (251, 658/251, 658)
NTT Hospital Group				
NTT East Japan Sapporo Hospital	99.99% (15, 841/15, 843)	100.00% (57, 062/57, 062)	100.00% (31, 577/31, 577)	100.00% (428, 411/428, 411)
Saga University Hospital	100.00% (15, 359/15, 359)	100.00% (58, 858/58, 858)	100.00% (41, 905/41, 905)	99.99% (511, 549/511, 577)
The University of Tokyo Hospital	100.00% (27, 439/27, 439)	100.00% (177, 077/177, 077)	100.00% (213, 939/213, 939)	100.00% (1, 729, 693/1, 729, 693)

*Note*. The data stored in MID‐NET^®^ were compared with the original electronic medical records in each hospital. Values in parentheses indicate the actual numbers of data for 1 month that were examined during a quality check, where the denominators are the original data numbers and the numerators are the stored data without errors. NTT East Japan Kanto Hospital contributed to this project in system management, but does not supply data. Therefore, the consistency check was carried out in the remaining 22 partner hospitals.

The processes described above also facilitate prompt root cause identification and data recovery when any issues are detected in MID‐NET^®^ data.

## 4 STANDARDIZED CODING PROCEDURES ACROSS ALL PARTNER HOSPITALS FOR INTEGRATED ANALYSES

As shown in Table 2, several coding standards are used to standardize EMR data in MID‐NET^®^ to allow the integration of data originating from different hospitals. In MID‐NET^®^, data based on localized codes used in each hospital are converted to these standardized codes while preserving the original clinical implication. To ensure the accuracy and uniformity of data coding across different hospitals, the PMDA collaborates with the partner hospitals to select the most appropriate codes (see Figure S2 for a detailed description of these procedures). During this process, a candidate code for each item is first selected by the PMDA on the basis of scientific rationale, and the applicability of the same codes to clinically identical data across hospitals is confirmed by the PMDA and the hospitals. If any differences in data or new localized codes are identified, discussions are held between the PMDA and the relevant hospitals to decide which code should be applied to the data. In the case of laboratory tests, the data distribution of each test is compared among the hospitals to consider the appropriateness of applying the codes across different hospitals. If an irregular case is identified, the PMDA contacts the relevant hospital to ascertain the reason for the irregularity and to find an appropriate solution. Similar to the development of the US Sentinel System,^13^ many discussions and analyses were required to choose the most appropriate coding standard for each laboratory test. Approximately 260 laboratory tests (eg, tests for liver function, renal function, and bone marrow function) were targeted for this mapping process (as of December 2018). The details for each laboratory test are available on the PMDA website (URL: http://www.pmda.go.jp/safety/mid-net/0001.html). More standardized tests will become available in the future after undergoing similar checks.

**Table 2:** Summary of coding standards used in MID‐NET^®^

Data Category	Data Type	Coding Standard	Outline
Diagnostic orders	Disease names	ICD‐10^a^	● Managed by the World Health Organization (WHO). ● Three to five digits: three digits (category code) + two digits (sequence code).
Prescription/injection orders	Drug names	YJ^b^, HOT^c^	<YJ> ● Managed by Iyaku‐Joho‐Kenkyujo, Inc. ● 12 digits: four digits (therapeutic category number) + three digits (route of administration + active ingredient) + one digit (dosage form) + one digit (different specification) + two digits (brand name) + one digit (check digit). <HOT> ● Managed by the Medical Information System Development Center (MEDIS). ● 13 digits: seven digits (prescription) + two digits (company) + two digits (dispensing package) + two digits (packaging quantity).
	Drug usage	JAMI usage standard^d^	● Managed by Japan Association for Medical Informatics (JAMI). ● 16 digits: one digit (basic usage) + one digit (detailed usage) + one digit (timing category) + 11 digits (administration timing in a day) + one digit (single/continuous dose and device) + one digit (home/hospital and self/professional‐administration).
	Dosage units	MERIT‐9^e^	● Managed by health level‐7 (HL‐7) Japan. ● Three to four digits for dosage forms and units.
Laboratory tests	Laboratory test names	JLAC10^f^	● Managed by the Japanese Society of Laboratory Medicine (JSLM). ● 17 digits: five digits (analyte code) + four digits (identification code) + three digits (specimen code) + three digits (methodology code) + two digits (result identification code).

a ICD‐10: International Statistical Classification of Diseases and Related Health Problems, Tenth Revision; https://www.who.int/classifications/icd/icdonlineversions/en/.

b YJ: Standard master (YJ codes) for pharmaceutical products; https://www.iyaku.info/yjcode/.

c HOT: Standard master (HOT codes) for pharmaceutical products; http://www2.medis.or.jp/master/hcode/.

d JAMI: Usage standard of the Japan Association for Medical Informatics; http://jami.jp/jamistd/.

e MERIT‐9: MEdical Record, Image, Text‐Information eXchange‐9 guidelines; http://www.jami.jp/jamistd/ssmix2.php.

f JLAC10: Japanese Laboratory Codes, Version 10; https://www.jslm.org/committees/code/.

In the case of administrative claims data and DPC data, the codes (eg, claims *processing system codes* and DPC codes) are standardized across hospitals based on the rules set by the government for the purpose of reimbursements. These administrative data represent the final data configuration that is actually used to determine reimbursements, and the codes are preserved in MID‐NET^®^ to reflect actual reimbursements. In addition, we confirmed that these data were appropriately sent to and stored in MID‐NET^®^ with high levels of data accuracy, consistency, and completeness.

## 5 VERIFICATION OF SYSTEM RELIABILITY

Inspections were performed to examine the reliability of the MID‐NET^®^ system in the data extraction process at each hospital, data transfer from each hospital to the central data center, and data conversion into the SAS^®^ format at the central data center. For example, the reliability of data extraction was confirmed through the following steps: (a) data were extracted from the MID‐NET^®^ database using a MID‐NET^®^ program, (b) the data were also manually and independently extracted from the database using the SAS^®^ program, and (c) reliability was examined by comparing the extracted data. Similar inspections were conducted for the other processes in the system. No major issues were detected during these inspections, which confirmed the reliability of the system.

## 6 CURRENT FEATURES OF MID‐NET^®^

Through the rigorous checks and analyses described above, MID‐NET^®^ was successfully established as a reliable and valuable medical information database. A general overview of MID‐NET^®^ and its advantages and limitations are summarized in Table 3. A major advantage of MID‐NET^®^ is the availability of many laboratory test results for analysis (approximately 260 tests; detailed lists are available on the PMDA website at http://www.pmda.go.jp/safety/mid-net/0001.html). For example, drug‐associated changes in liver, renal, or bone marrow function can be measured directly through the use of relevant parameters from the laboratory test results. MID‐NET^®^ is also designed to fulfill the requirements of GPSP and their related guidelines.^10^, ^11^ Accordingly, the pharmaceutical industry is able to utilize MID‐NET^®^ to provide post‐marketing surveillance data for regulatory submission in Japan. The general characteristics of MID‐NET^®^ indicate that data for analysis are available across a broad range of patient ages and diseases, as well as a wide variety of prescription drugs (see Figure S3 for more details). However, the partner hospitals generally comprise mid‐sized and large hospitals, such as university hospitals and regional core hospitals. Therefore, the following trends may be observed in MID‐NET^®^ data when compared with a general patient population in Japan^14^: (a) a lower proportion of very elderly patients, who may be more likely to visit a nursing care hospital or rehabilitation hospital than a MID‐NET^®^ partner hospital; (b) a higher proportion of patients with acute and severe conditions, which would be seen in some diseases such as infectious diseases and cancer; and (c) a lower proportion of treatments that are usually provided by general practitioners or clinics, such as treatments for mental disorders, dental care, and preventive vaccinations. In addition, as of December 2018, there are data from approximately 4.7 million patients in the MID‐NET^®^ database, with an increase of approximately 0.5 million patients expected every year. Given that the Japanese population comprises almost 127 million people, this sample size may still be fairly small. In particular, MID‐NET^®^ may have limited applications for the analysis of rare diseases and orphan drugs. Another limitation is that data cannot be linked across hospitals when a patient moves from one hospital to another. These points should be taken into consideration when evaluating data in terms of the generalizability of analytical results based on MID‐NET^®^ data. We have recently reported the results of pilot pharmacoepidemiological studies using MID‐NET^®^ data for drug safety assessments.^15^ These studies can help to promote an understanding of the characteristics and appropriate analysis of MID‐NET^®^ data.

**Table 3:** General information and advantages and limitations of MID‐NET^®^

General Information	
The partner hospitals	10 organizations including 23 mid‐sized and large hospitals
Number of subjects	Approximately 4.7 million patients as of December 2018
Finance	All expenses from the Ministry of Health, Labour and Welfare and Pharmaceuticals Medical Devices Agency
Collected target data	EMRs, claims, and DPC data

Advantages	Limitations
✓ High data quality ➢Daily and periodic quality checks are conducted. ➢Accuracy, consistency, and completeness between extracted and original data is periodically confirmed. ✓ Frequent updates of stored data. ➢Data are updated every week or 1‐3 months. ✓ Wide variety of data, including EMRs data, claims data, and DPC data. ➢Data categories can be linked at the patient level. ✓ Detailed checks for approximately 260 standardized laboratory tests have been conducted. ➢Reliability of coding has been confirmed. ✓ Regulatory requirements are met. ➢The requirements of regulatory standards (“good post‐ marketing study practice” standards) have been fulfilled. ➢Can be used as a major source of data in regulatory assessments of drug safety.	✓ Sample size is still relatively small. ✓ No patient‐level linkage of data among hospitals. ➢Loss of follow‐up when a patient moves across hospitals. ✓ Only medium‐to‐large hospitals are represented. ➢Mainly university hospitals and regional core hospitals without general practitioner and clinics. ➢Higher proportion of patients with acute and severe conditions.

Abbreviations: DPC, diagnosis procedure combination; EMRs, electronic medical records.

## 7 CHALLENGES IN ESTABLISHING A RELIABLE AND VALUABLE DATABASE FOR DRUG SAFETY ASSESSMENTS

On the basis of our experiences in the development of MID‐NET^®^, we found that consistent data quality management was vital to establishing a reliable and valuable database that has applications in regulatory science. Furthermore, hospital‐level differences in the actual management and interpretation of coding standards for health and billing records should be taken into consideration to ensure data quality and reliability. The creation of an organizational cultural environment that supports synergistic collaborations among all involved parties (including the partner hospitals, the MHLW, the PMDA, and associated information technology companies) was also crucial to the success of this project. The experiences and best practices established in MID‐NET^®^ may provide a model for the future development of similar database networks.

Since its inauguration on April 2018, MID‐NET^®^ still faces many challenges, especially with regard to data quality maintenance. Periodic data quality checks will be necessary to confirm that newly stored data are consistent with the original data, and its local codes are appropriately converted to the standardized codes while preserving the original clinical implication. In particular, the coding procedure should be timely and sustainable to ensure that the most recent data are available for integrated analysis of data from different partner hospitals. In addition, quality assurance is an indispensable prerequisite for allowing the utilization of real‐world data for regulatory purposes. Another major challenge is the future expansion of MID‐NET^®^ without any loss in data quality. The Japanese government is considering the possibility of linking MID‐NET^®^ with other databases to promote the utilization of real‐world data in Japan.^16^, ^17^ Since MID‐NET^®^ is a complex distributed database that requires substantial resources, it would be necessary to establish an efficient and feasible process to manage and maintain data quality amidst an increasing number of partner hospitals and the formation of linkages with other databases. The utilization of real‐world data for regulatory purposes is still in its learning phase, and international regulatory collaborations (with an emphasis on experience sharing and common understanding) will be needed to promote international integration and advance regulatory science. We will continue to work to further the development of MID‐NET^®^ as an internationally recognized medical information database network for assessing and improving drug safety.

## FUNDING INFORMATION

This was a government project that received no external funding.

## CONFLICT OF INTEREST

The authors have no financial or personal relationships with other people or organizations that could inappropriately influence or bias the content of this paper.

## ORGANIZATIONS AND MEMBERS OF MID‐NET^®^ PROJECT

### Pharmaceuticals and Medical Devices Agency (PMDA)

Takashi Ando, Ayumi Endo, Daisuke Fujii, Sayoko Harada, Tomoaki Hasegawa, Kohei Hayashi, Koshi Hichiwa, Shingo Higa, Kaori Hirata, Wakako Horiki, Yukiko Hosaka, Mari Hosoda, Yasuo Iimura, Mie Ikeda, Kazutaka Imao, Yoshihiko Inazumi, Satomi Inomata, Chieko Ishiguro, Maori Ito, Eiko Iwasa, Takuya Kageyama, Kazuhiro Kajiyama, Ken Kakihara, Maiko Kawaguchi, Sanmai Ki, Mari Kishiba, Yutoku Kitahara, Chizuru Kobayashi, Mei Kohama, Maki Komamine, Emiko Kondo, Aiko Kumano, Yuta Maeda, Emi Masumitsu, Bunpei Matoba, Kazuhiro Matsui, Yu Matsuzaki, Mai Miyaoka, Satomi Nagashima, Satoru Nakamura, Atsushi Noguchi, Maki Noguchi, Michihiro Ogawa, Hiroyuki Okuda, Souno Sawada, Miyuki Sunohara, Chiho Suzuki, Kenji Suzuki, Fumitaka Takahashi, Kanako Takasaki, Yoshinori Takeuchi, Ayumi Tanaka, Kento Tanaka, Kotaro Tomita, Mayuko Ujibe, Yoshiaki Uyama, Kenichi Watanabe, Shinichi Watanabe, Kaori Yamada, Risa Yamada, Mitsune Yamaguchi

### Collaborative organizations

**Chiba University Hospital:** Shunsuke Ito, Takashi Kimura, Kenichiro Shimai, Takahiro Suzuki

**Hamamatsu University Hospital**: Michio Kimura, Satoru Ono (The University of Tokyo), Hiroshi Watanabe (National Centre for Geriatrics and Gerontology)

**Kagawa University Hospital**: Yoko Kataoka, Jun Kunikata, Tomomichi Subgawara, Hideto Yokoi

**Kitasato Institute Group**: Koichiro Murata

**Kyushu University Hospital**: Naoki Nakashima, Takanori Yamashita, Yoshifumi Wakata, Yasunobu Nohara, Tadashi Kandabashi, Rieko Izukura, Jinsang Park, Chinatsu Nojiri, Atsushi Takada, Taeko Hotta, Dongchon Kang, Satohiro Masuda

**NTT hospital group**: Takao Orii

**Saga University Hospital**: Eizaburo Sueoka

**Tohoku University Hospital**: Ryusuke Inoue, Masaharu Nakayama, Chiaki Otomo

**Tokushukai Group**: Yoshiaki Fujimura, Hiroko Nomura

**The University of Tokyo Hospital**: Tatsuo Hiramatsu, Kazuhiko Ohe, Katsuya Tanaka

## Supporting information

Figure S1. Outline of data anonymization process in MID‐NET®

Figure S2. General process and communications between PMDA and a partner hospital to apply the appropriate standard codes

Figure S3. General characteristics of MID‐NET® data
