# Supplementary figures and images for: Establishment of the MID‐NET® medical information database network as a reliable and valuable database for drug safety assessments in Japan
Source: Pharmacoepidemiol Drug Saf. 2019 Aug 29;28(10):1395–404. doi: 10.1002/pds.4879 (PMC6851601; doi:10.1002/pds.4879)

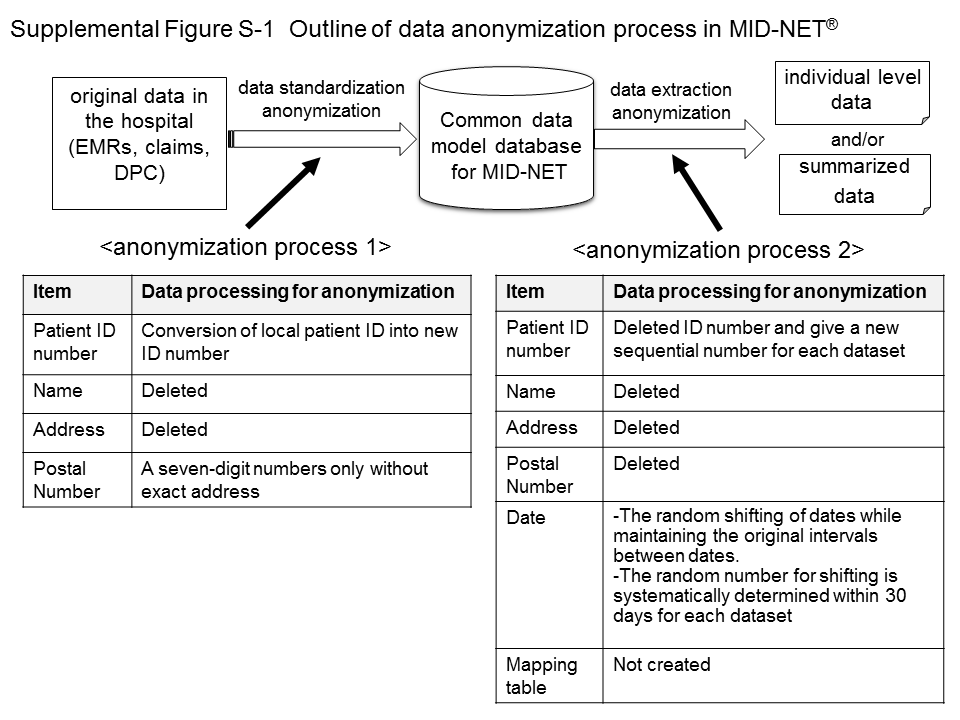

Supplement: Supplementary file 1 — Figure S1. Outline of data anonymization process in MID‐NET® [file PDS-28-1395-s001.tif]

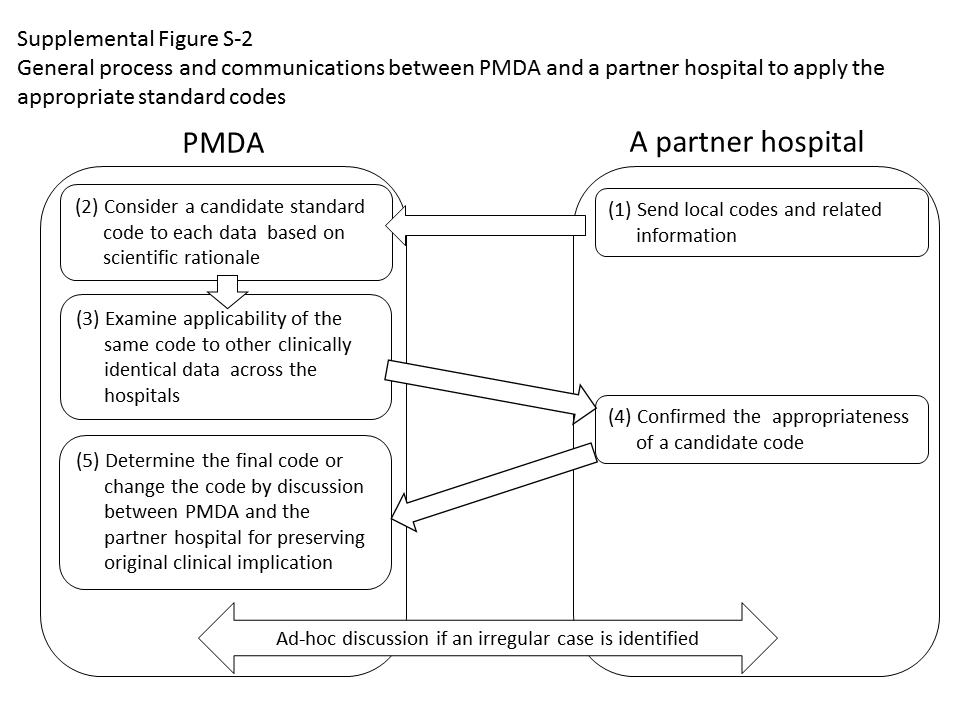

Supplement: Supplementary file 2 — Figure S2. General process and communications between PMDA and a partner hospital to apply the appropriate standard codes [file PDS-28-1395-s002.tif]
